# Supplementary material for: A versatile genetic tool for post-translational control of gene expression in Drosophila melanogaster
Source: eLife. 2017 Nov 15;6:e30327. doi: 10.7554/eLife.30327 (PMC5703639; doi:10.7554/eLife.30327)
Supplement: Supplementary file 1. [file elife-30327-supp1.docx]

Table1:Genotypes and feeding condition by experiment

| **Figure** | **Genotype** | **Feeding condition** |
| --- | --- | --- |
| 1B | *w- ; + ; nsyb-GAL4/10XUAS-GFP-DD* | Flies fed with standard fly food or food containing 1 mM TMP from embryo stage to adult up to dissection |
| 1C, D | *w- ; + ; Orco-GAL4/10XUAS-GFP-DD* | Flies fed with standard fly food containing 0 – 5 mM TMP for 48 hours |
| 1E, F | *w- ; + ; Orco-GAL4/10XUAS-GFP-DD* | Flies fed with standard fly food containing 1 mM TMP for 0 - 60 hours |
| 1G, H | *w- ; + ; Orco-GAL4/10XUAS-GFP-DD* | Flies fed with standard fly food containing 1 mM TMP for 48 hours, and then moved to standard fly food without TMP for 0-36 hours |
| 1-S2A | *w- ; + ; nsyb-GAL4/10XUAS-GFP-DD* | Flies fed with standard fly food or food containing 1 mM TMP from embryo up to dissection. |
| 1-S3A | Sugar gustatory neurons:  *w- ; + ; Gr5a-GAL4/10XUAS-GFP-DD*  Olfactory receptor neurons:  *w- ; + ; Orco-GAL4/10XUAS-GFP-DD*  PAM dopamine neurons:  *w- ; + ; R58E02-GAL4/10XUAS-GFP-DD*  P1a neurons: *w- ; R15A01-AD/+ ; R71G01-DBD /10XUAS-GFP-DD*  MB output neurons: *w- ; R30E08-p65AD/+; R53C10-DBD*  */10XUAS-GFP-DD*  Olfactory projection neurons: *w- ; GH146-GAL4/+; 10XUAS-GFP-DD/+*  Aminergic neurons:  *w- ; Tdc2-GAL4/+; 10XUAS-GFP-DD/+*  Kenyon cells:  *w- ; + ; MB247-GAL4/10XUAS-GFP-DD* | Flies fed with standard fly food or food containing 1 mM TMP from embryo up to dissection. |
| 1-S3B,C | *w-; Actin5C-GAL4/+ ; 10XUAS-GFP- DD/+* | Flies fed with 1 mM TMP or 1% DMSO in standard fly food for 48 hours before dissection. |

| 1-S3D | *w-; + ; Orco-LexA/13xLexAop-GFP-DD/+* | Flies fed with 1 mM TMP or 1% DMSO in standard fly food for 48 hours before dissection. |
| --- | --- | --- |
| 1-S4A | Wild-type (w1118) | Flies fed with 0 -10 mM TMP in 1 M sucrose, 1% agar from eclosion to death |
| 1-S4B | Wild-type (w1118) | Flies raised in 0-10 mM TMP in standard fly food from embryo onwards. |
| 1-S4C | Wild-type (Canton-S) | Flies fed with 1 mM TMP or 1% DMSO in standard fly food for 48 hours before behavioral assay. |
| 1-S4D | Wild-type (Canton-S) | Flies fed with 1 mM TMP or 1% DMSO in standard fly food for 24 hours, following which flies were transferred to starvation vials containing water with 1 mM TMP or 1% DMSO for 24 hours before behavioral assay. |
| 2A | *w- ; UAS-GFP/+ ;*  *Orco-GAL4/nsyb-GAL80-DD* | Flies fed with standard fly food or food containing 1 mM TMP from embryo stage to adult up to dissection |
| 2B, C | *w- ; UAS-GFP/+ ;*  *Orco-GAL4/nsyb-GAL80-DD* | B1, C1: Flies fed with food containing 1mM TMP from embryo stage to 3 days post eclosion. Flies were then transferred to standard food without TMP up to dissection.  B2, C2: Flies fed with standard food from embryo stage to adult up to dissection.  B3, C3: Flies fed with food containing 1mM TMP from embryo to adult up to dissection. |
| 2D, E | *w-/+ ; Gr21a-GAL4/+ ; +*  *w-/+ ; UAS-TNT/+ ; nsyb-GAL80-DD/+*  *w-/+; Gr21a-GAL4, UAS-TNT/+; nsyb- GAL80-DD/+* | Flies fed with standard fly food or food containing 1 mM TMP from embryo stage to adult up to behavioral assay |
| 2-S1B,C | *w- ; UAS-GFP/+ ;*  *Orco-GAL4/nsyb-GAL80-DD* | Flies raised on food containing 1 mM TMP from embryo stage up to eclosion. Flies were then transferred to standard food without TMP (B, top) or maintained on food containing 1 mM TMP (B, bottom). |
| 3A, B | *w- ; GH146-GAL4/+ ;*  *UAS(FRT.STOP)mCD8GFP/ 10XUAS- FLP-DD* | Flies fed with standard fly food containing 0 – 1 mM TMP from embryo stage up to dissection. |

| 3C,D | *w- ; GH146-GAL4/+ ; UAS(FRT.STOP)mCD8GFP/ 10XUAS- FLP-DD* | Flies fed with standard fly food without TMP from embryo stage up to dissection. |
| --- | --- | --- |
| 4A | *w-; GH146-QF/UAS-6xmcherry-HA; NP21-GAL4/QUAS-6xGFP* | Flies fed with standard fly food from embryo stage up to dissection |
| 4B | *w-; GH146-QF/ QUAS(FRT.STOP)mCD8GFP ; NP21-GAL4/ UAS-FLP* | Flies fed with standard fly food from embryo stage up to dissection |
| 4C | *w-; GH146-QF/ UAS(FRT.STOP)GFP.myr; NP21-GAL4/ QUAS-FLP* | Flies fed with standard fly food from embryo stage up to dissection |
| 4D-F | *w-; GH146-QF/ QUAS(FRT.STOP)mCD8GFP ; NP21-GAL4/ UAS-FLP-DD* | D: Flies fed with standard fly food without TMP from embryo stage up to eclosion. After eclosion, flies were switched to food containing 1 mM TMP for 4-5 days before dissection.  E: Flies fed with standard fly food from embryo stage up to dissection  F: Flies fed with fly food containing 1mM TMP from embryo stage up to dissection. |
| 4-S1 | *w-; GH146-QF/ QUAS(FRT.STOP)mCD8GFP ;*  *NP21-GAL4/ UAS-FLP-DD* | Flies fed with fly food containing 10 mM TMP from embryo stage up to dissection. |
